# Whole genome sequencing of Avian metapneumovirus type B genomes directly from clinical samples collected from chickens in live bird markets using multiplex tiling RT-PCR method

**DOI:** 10.3389/fvets.2023.1112552

**Published:** 2023-03-02

**Authors:** Andrew Y. Cho, Tae-Hyeon Kim, Sun-Hak Lee, Heesu Lee, Yun-Jeong Choi, Ye-Ram Seo, Dong-Hun Lee, Ji-Yeon Hyeon, Chang-Seon Song

**Affiliations:** ^1^Avian Disease Laboratory, College of Veterinary Medicine, Konkuk University, Gwangjin-gu, Seoul, Republic of Korea; ^2^Wildlife Health Laboratory, College of Veterinary Medicine, Konkuk University, Seoul, Republic of Korea; ^3^KHAV Co. Ltd., New Millennium Hall, Gwangjin-gu, Seoul, Republic of Korea

**Keywords:** Avian metapneumovirus (aMPV), chickens, live bird market, clinical samples, whole genome sequencing

## Introduction

Avian metapneumovirus (AMPV), a member of the family *Pneumoviridae*, genus *Metapneumovirus* possesses a non-segmented negative-sense RNA genome of approximately 13–15 kb with eight genes (3′-N-P-M-F-M2-SH-G-L-5′) (1– 3). AMPV causes rhinotracheitis in turkeys and swollen head syndrome (SHS) in chickens, mostly contributing to secondary bacterial infections leading to more severe symptoms in chickens (4).

Different types of AMPVs have been classified based on the nucleotide sequence divergence of the attachment glycoprotein (G) and antigenic differences between strains (5). Types A and B are found all around the world, and type C was reported in North America, China, and South Korea, and in a retrospective study in France in 1990 (6). Type D was reported only once in a turkey flock in France in 1985 (5, 7). AMPV types A, B, and C have been detected in South Korea; the type A and B in poultry farms and the type C from pheasants in live bird markets (6, 8, 9).

The study of AMPV infection is particularly difficult due to the transient nature of viral shedding in the host before the symptoms develop. In most cases, multiple blind passages of AMPV from the clinical samples are required for isolation and identification (4, 5). Therefore, a limited number of AMPV sequences are available in the NCBI GenBank database. As of November 29, 2022, there are only three complete genome sequences of AMPV type B (LN16, VCO3/60616, and Hungary/657/4; MH745147, AB548428, and MN729604 respectively) (3, 10, 11).

The tiling amplicon method has been proven to be efficient and prolific in producing whole genome sequences directly from clinical samples during the Covid-19 pandemic (12). In a previous study, the multiplex tiling RT-PCR method was applied to enrich the genetic material of Zika virus and Rabies lyssavirus directly from brain tissue samples, and it enables to sequence of the full genome of low titer samples containing as low as 50 genome copies in a reaction (13, 14).

In this study, we detected 6 AMPVs from chickens in live bird markets (LBM) in South Korea during 2019–2022. For whole genome sequencing of AMPVs, we developed a PCR primer panel to efficiently amplify the complete coding region of AMPV type B using the multiplex tiling RT-PCR method. We successfully obtained the complete coding region of AMPVs using Illumina next-generation sequencing (NGS) and conducted comparative a phylogenetic analysis to analyze the genetic relatedness of AMPVs from LBMs in Korea with other AMPVs.

## Materials and methods

### Sample collection

A total of 138 slaughtered chickens were purchased from poultry meat vendors in LBMs in Korea during the period of 2019 to 2022. The nasal turbinate or whole beaks of the chickens were collected and washed with phosphate-buffered saline (PBS) using enough to fully immerse the samples. Total RNA was extracted from the nasal turbinate wash samples using the Qiagen RNeasy mini kit (Hilden, Germany) and used to detect both Avian metapneumovirus type A and B using the real-time qRT-PCR as previously described (8). All positive nasal turbinate wash samples were inoculated to Vero cells for virus isolation.

### Primer design and tiling amplicon PCR

To design a tiling amplicon PCR primer panel, full genome sequences of AMPV type B available in the NCBI GenBank (LN16, VCO3/60616, and Hungary/657/4; Accession no. MH745147, AB548428, and MN729604 respectively) were downloaded and aligned using the MAFFT 1.4.0 program on Geneious Prime software (https://www.geneious.com) (3, 10, 11). Primers were designed to amplify 380–420 bp region with about 100 bp overlap, and 43 sets of designed primers were pooled into two pools according to the primer design output by Primal Scheme (http://primalscheme.com) (Supplementary Table 2) (13). Three of six positive samples with lower cycle threshold (Ct) values (ranging from 25.77 to 30.6) were selected for subsequent multiplex RT-PCR (Table 1). cDNA was synthesized from the RNA using the LunaScript RT SuperMix kit (NEB, Massachusetts, United States) following the manufacturer's instructions. For the multiplex PCR assay, an equal volume of each 100 uM primer stock was pooled together as designated as pool 1 and 2 (Supplementary Table 2). The PCR mixture was prepared by mixing 12.5 ul Q5 Hotstart 2X Master Mix (NEB, Massachusetts, United states), 0.015 uM of each primer pool, 5 ul of template cDNA, and nuclease-free water up to 25 ul. The reaction mixture was prepared for each pool as it induces consistency between reactions and PCR assay was performed. PCR amplification conditions were: 98°C for 15 s, followed by 35 cycles of 95°C for 15 s and 63°C for 5 min (12). The PCR products were then visualized by electrophoresis on 2% agarose gels showing around 400 bp amplicons (13). PCR products of both primer pools were combined and used for library preparation after purification using the Qiaquick PCR purification kit (Qiagen, Hilden, Germany). Library was prepared using the TruSeq Nano DNA kit (Illumina, California, United States) to produce 2 × 151 bp paired-end reads. Library preparation and sequencing on NextSeq 500 sequencing system (Illumina, California, United States) was done by LAS (Gimpo, Republic of Korea).

**Table 1:** Genome sequencing and assembly results of Avian metapneumovirus type B isolates from this study.

**Isolates**	**Real-time PCR (Ct value^a^)**	**Total NGS reads**	**Trimmed reads (>Q20)**	**Genome assembly results**		
				**Number of assembled reads**	**Coverage**	**Complete CDS**
AMPV/B/Korea/N19-29/2019	28.67	9,016,084	8,632,844	7,925,758 (91.8%)^b^	99.3% (13,414 of 13,513)	Yes
AMPV/B/Korea/N21-41/2021	25.77	8,427,438	8,178,950	7,563,434 (92.5%)	99.2% (13,411 of 13,513)	Yes
AMPV/B/Korea/N21-83/2021	30.6	7,867,590	7,609,232	6,967,759 (91.6%)	98.3% (13,279) of (13,513)	No [Gaps in F (5 bp) and L (100 bp)]^c^

^a^Cycle threshold values of real-time RT-PCR.

^b^Percentage of mapped reads/trimmed reads.

^c^Gaps were filled with two sanger sequencing reads produced with sample PCR primers used for multiplex RT-PCR.

### Assembly and phylogenetic analysis

Raw reads were trimmed of adapters and low-quality bases using BBDuk version 38.84 by setting the minimum quality to 20 (15). *De novo* and reference-based assemblies of genome sequences were performed. For reference-based assembly, trimmed reads were mapped to the LN16 virus genome (GenBank accession number: MH745147) using the Minimap 2.24 (https://github.com/lh3/minimap2) with default options and visualized on Geneious Prime software. Trimmed reads were assembled *de novo* using the SPAdes assembler 3.15.5. The assembled genome sequences produced by reference mapping and *de novo* assembly approaches were combined to generate the final consensus genome sequences. A total of 48 G sequences of AMPV type B were downloaded from the GenBank database and aligned using the MAFFT Multiple Sequence Alignment software v7.450 for phylogenetic analysis (16). A maximum-likelihood (ML) phylogenetic tree was reconstructed using the RAxML GUI 2.0 (https://antonellilab.github.io/raxmlGUI/) with a rapid bootstrap option set to 1,000 (17).

## Descriptive results

A total of 6 out of 138 (4.34%) chicken nasal turbinate wash samples tested positive for AMPV type B by real-time qRT-PCR (Supplementary Table 1 and Supplementary Figure 1). Sequencing results confirmed that the positive samples from the surveillance were not a result of contamination as the detected APMV sequences differ from each other and the rest of the APMV genomes found in the GenBank database. The inoculated Vero cells did not exhibit any cytopathic effect, suggesting it may require additional passages for virus isolation (data not shown). We successfully obtained complete coding genome sequences (CDS) of all three nasal turbinate samples (AMPV/B/Korea/N19-29/2019, AMPV/B/Korea/N21-83/2021, and AMPV/B/Korea/N21-41/2021; hereafter N19-29, N21-83, and N21-41, respectively) (Table 1). Our tiling amplicon PCR reactions showed a fairly small amplification bias when clinical samples with relatively low Ct values, 25.77 and 28.67, were used. We were able to assemble complete CDS of N19-29 and N21-41 viruses using the tiling amplicon PCR primer panel designed in this study coupled with illumina NGS. However, the N21-83 virus which showed a higher Ct value (30.6) had two short gaps in the initial genome assembly results, including 5 bp in F gene and 100 bp in L gene. The gaps were covered with two sanger sequencing reads using the same primers used in this study. Since the two fragments were amplified using the same primers, the outcome of low amplification efficiency was not likely due to primer-template mismatch. We assume that competitive inhibition between primers may have decreased PCR efficiency of the fragments.

**Figure 1:**
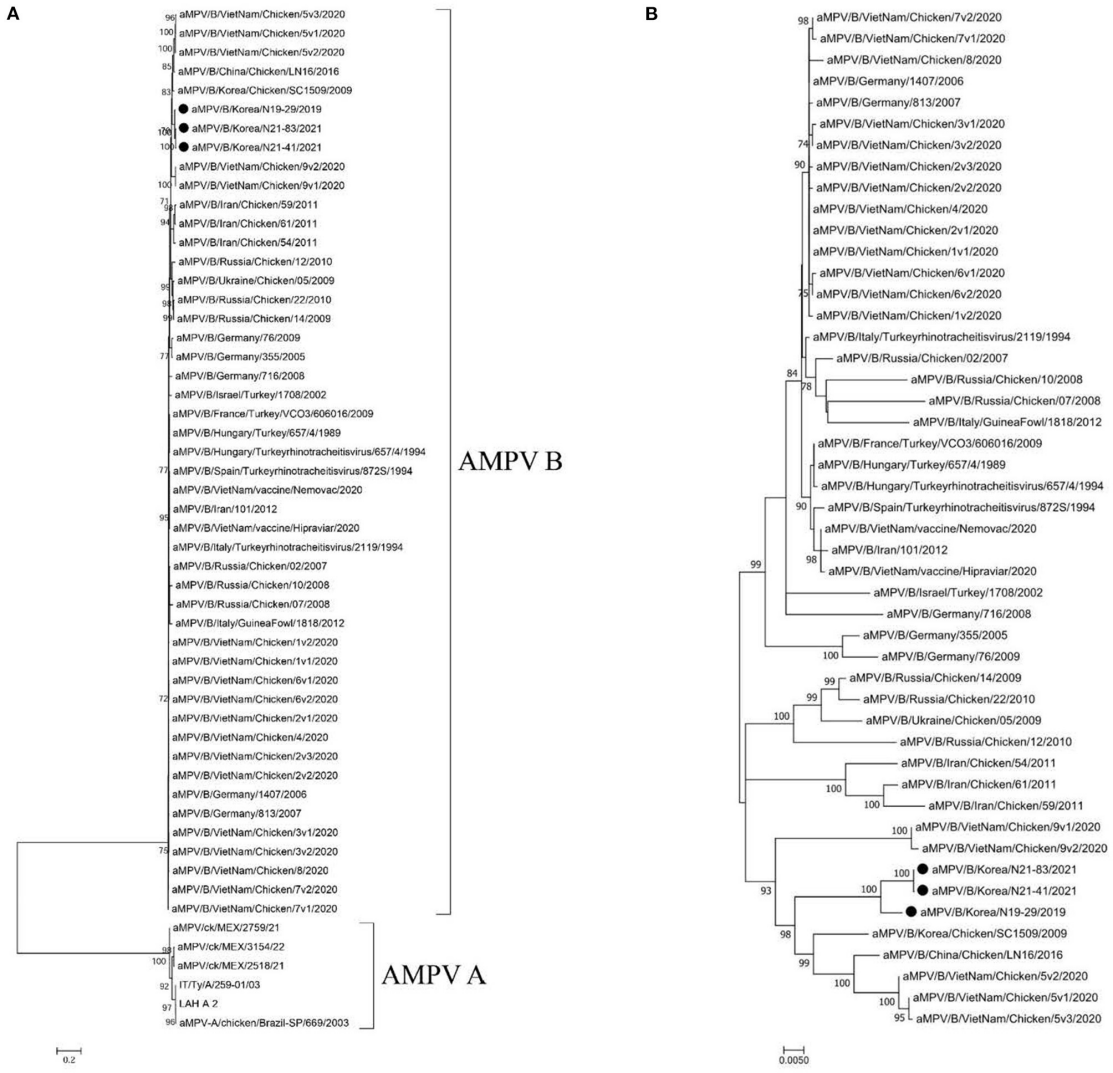
**(A)** Maximum-likelihood analysis of 54 glycoprotein (G) sequences of AMPV type A and B. It is rooted to the AMPV type A sequences and **(B)** Maximum-likelihood analysis of 48 glycoprotein (G) sequences of AMPVs type B downloaded from GenBank database, rooted to midpoint. Closed circles (•) indicate the viruses sequenced in this study. The scale bars show the number of substitutions per site. The numerical values represent 1,000 bootstrap replicate values expressed as a percentage.

Phylogenetic analysis of G gene sequences revealed that the viruses sequenced in this study belong to the AMPV type B and showed the closest genetic relationship with the SC1509 (GenBank Accession no. DI187010.1) virus (nucleotide sequence identity: 95.74–96.14%) from Korea in 2009 and the LN16 virus (nucleotide sequence identity: 95.42–95.82%) identified from China in 2016 (Figure 1). The N19-29, N21-83, and N21-41 viruses did not cluster with the commercial live attenuated AMPV type B vaccine strains in phylogenies (Figure 1). We were not able to present phylogenetic analysis based on whole-genome sequences as currently there are only 3 complete genome sequences of AMPV type B available in the NCBI GenBank database.

In conclusion, we developed the tiling amplicon PCR method for genome sequencing and successfully sequenced three AMPVs directly from clinical samples. The multiplex tiling RT-PCR and NGS approach developed in this study has the potential to be implemented in a diagnostic setting, providing a rapid and reliable method for complete genome sequencing and molecular epidemiological study of AMPV from clinical samples. In addition, the complete CDS of AMPVs established in this study would be useful as reference data for future investigations on AMPVs. The relative ease of acquiring complete CDS directly from clinical samples without a labor-intensive adaptation process in cell culture will help further the diversity of the AMPV genome database. Full genome sequencing of AMPV have suggested subpopulation present in vaccine strain could be selected for better replication during *in vivo* replication in field conditions (18). Our method could also be used to monitor mutations and subpopulations of field strains without prior adaptation, allowing for more accurate representation of AMPV quasi-species. In addition, since the live bird markets have been recognized as a reservoir, amplifier, and source of Avian viruses (19), genomic surveillance of AMPVs in LBMs should be enhanced for monitoring of further evolution and spread of the AMPVs.

## Data availability statement

The datasets presented in this study can be found in online repositories. The names of the repository/repositories and accession number(s) can be found below: https://www.ncbi.nlm.nih.gov/genbank/, OP924005, OP924006, and OP924007. https://www.ncbi.nlm.nih.gov/sra, SRR22993455, SRR22993456, and SRR22993457.

## Author contributions

Conceptualization and data analysis: AC. Methodology, data curation, and writing—original draft preparation: AC and T-HK. Sample preparation: AC, T-HK, S-HL, HL, Y-JC, and Y-RS. Writing—review and editing: D-HL, J-YH, and C-SS. Supervision and funding acquisition: J-YH and C-SS. All authors have read and agreed to the published version of the manuscript.
