# Supplementary material for: Whole genome sequencing of Avian metapneumovirus type B genomes directly from clinical samples collected from chickens in live bird markets using multiplex tiling RT-PCR method
Source: Front Vet Sci. 2023 Mar 2;10:1112552. doi: 10.3389/fvets.2023.1112552 (PMC10018015; doi:10.3389/fvets.2023.1112552)
Supplement: Supplementary file 1 [file Data_Sheet_1.PDF]

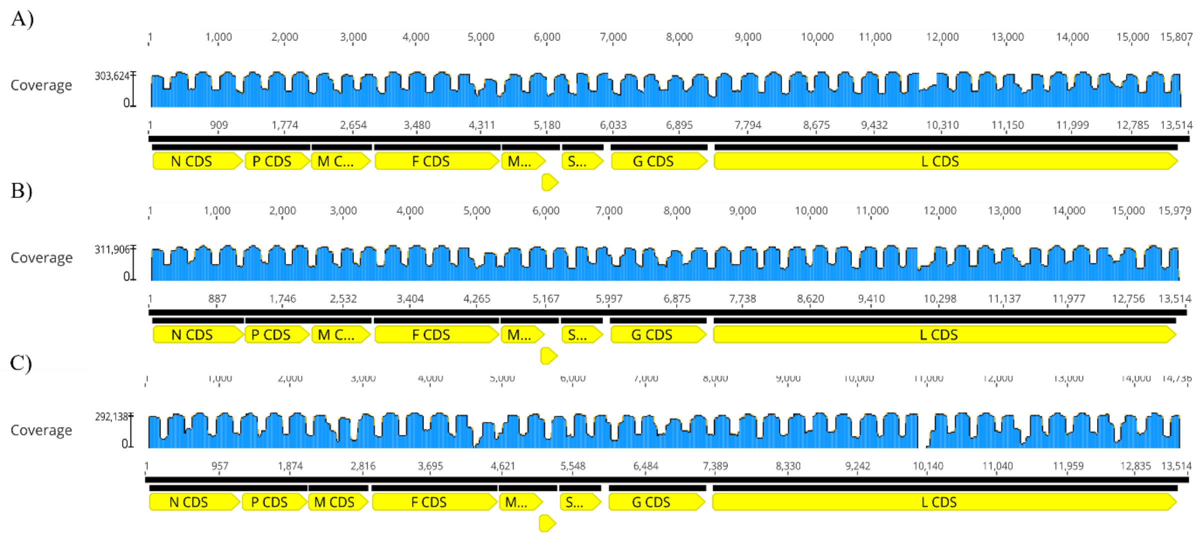

Supplementary Figure S1. Coverage plots of (A)AMPV/B/Korea/N1929/2019, (B)AMPV/B/Korea/N21-41/2021, and (C)AMPV/B/Korea/N21-83/2021 visualized using Geneious Prime software. The x-axis represents the length of the genome (13,513 nucleotides). The y-axis represents the sequencing depth on a logarithmic scale.
