# Supplementary material for: Whole genome sequencing of Avian metapneumovirus type B genomes directly from clinical samples collected from chickens in live bird markets using multiplex tiling RT-PCR method
Source: Front Vet Sci. 2023 Mar 2;10:1112552. doi: 10.3389/fvets.2023.1112552 (PMC10018015; doi:10.3389/fvets.2023.1112552)
Supplement: Supplementary file 2 [file Table_1.docx]

**Supplementary Table S1.** Surveillance results of avian metapneumoviruses (AMPV) from chickens in live bird market.

| **Year** | **No. of collected samples** | **No. of positive samples** | |
| --- | --- | --- | --- |
|  |  | **AMPV Type A** | **AMPV Type B** |
| 2019 | 35 | 0 | 3 |
| 2020 | 7 | 0 | 0 |
| 2021 | 84 | 0 | 3 |
| 2022 | 12 | 0 | 0 |
