# Supplementary material for: Whole genome sequencing of Avian metapneumovirus type B genomes directly from clinical samples collected from chickens in live bird markets using multiplex tiling RT-PCR method
Source: Front Vet Sci. 2023 Mar 2;10:1112552. doi: 10.3389/fvets.2023.1112552 (PMC10018015; doi:10.3389/fvets.2023.1112552)
Supplement: Supplementary file 3 [file Table_2.docx]

**Supplementary Table S2.** Primer panel for the genome sequencing of AMPV type B designed by using the Primal Scheme software.

| **Primer** | **Pool** | **Sequence** | **Primer**  **Size (bp)** | **%GC** | **TM (℃)** |
| --- | --- | --- | --- | --- | --- |
| AMPV_B_400_1_LEFT | 1 | TGGGACAAGTAAAAATGTCTCTTGAA | 26 | 34.62 | 59.61 |
| AMPV_B_400_1_RIGHT | 1 | CGTTCCACATTGTCTGCCCAT | 21 | 52.38 | 61.32 |
| AMPV_B_400_2_LEFT | 2 | CTGCAGAAGGTCCCATACGAAA | 22 | 50 | 60.54 |
| AMPV_B_400_2_RIGHT | 2 | AGACGTACTCCCAAGTGCCTTA | 22 | 50 | 61 |
| AMPV_B_400_3_LEFT | 1 | GATTGCCAAATCCTTCTTTGAGCT | 24 | 41.67 | 60.4 |
| AMPV_B_400_3_RIGHT | 1 | TGCTCTGCCCTTATACATCCCA | 22 | 50 | 61.15 |
| AMPV_B_400_4_LEFT | 2 | ACTATCATTAACAAGTTGCCCCAAC | 25 | 40 | 60.2 |
| AMPV_B_400_4_RIGHT | 2 | TGTGCTAACAGGGTCACCACTA | 22 | 50 | 61.21 |
| AMPV_B_400_5_LEFT | 1 | AAGTTGGCAGAGGCTTATCAGC | 22 | 50 | 61.13 |
| AMPV_B_400_5_RIGHT | 1 | TGTCCTTCTCCTCAAATGTAAGTACA | 26 | 38.46 | 60.18 |
| AMPV_B_400_6_LEFT | 2 | TAGAAGAAGAAGCCCTGGAGCT | 22 | 50 | 60.75 |
| AMPV_B_400_6_RIGHT | 2 | CTCGAGCTTTGATCCTCAAGCA | 22 | 50 | 60.86 |
| AMPV_B_400_7_LEFT | 1 | GCACAGAGGGCAAAAATAGGAG | 22 | 50 | 59.75 |
| AMPV_B_400_7_RIGHT | 1 | CTTGTAGCACTGGTCCTTCAGG | 22 | 54.55 | 60.79 |
| AMPV_B_400_8_LEFT | 2 | ACTCCTGCTCCAGTGCTACT | 20 | 55 | 60.27 |
| AMPV_B_400_8_RIGHT | 2 | GCTTCAACTGTAGCTGACTCGG | 22 | 54.55 | 61.42 |
| AMPV_B_400_9_LEFT | 1 | AACAAACATGAACACCGTTGGG | 22 | 45.45 | 60.34 |
| AMPV_B_400_9_RIGHT | 1 | AATAGTGGACTTTTGTTTGCCAGT | 24 | 37.5 | 59.67 |
| AMPV_B_400_10_LEFT | 2 | AGGCTGAGAGCTTAGGGAAAATATG | 25 | 44 | 60.84 |
| AMPV_B_400_10_RIGHT | 2 | TCTCCTTTGTAATCTGATCGGCA | 23 | 43.48 | 59.8 |
| AMPV_B_400_11_LEFT | 1 | TGGTCCTAGCCTTATCAGCACT | 22 | 50 | 60.81 |
| AMPV_B_400_11_RIGHT | 1 | TTCTGTCCAAAGCTGATTGCCA | 22 | 45.45 | 60.94 |
| AMPV_B_400_12_LEFT | 2 | AGCAAGAAGTTAACCCCTGCAA | 22 | 45.45 | 60.61 |
| AMPV_B_400_12_RIGHT | 2 | AGCAGACAAGCATAACTCTCCC | 22 | 50 | 60.28 |
| AMPV_B_400_13_LEFT | 1 | TGGAGTTATAGAGACACCCTGCT | 23 | 47.83 | 60.57 |
| AMPV_B_400_13_RIGHT | 1 | TGTGTGTGCATCCTTTGTTGAG | 22 | 45.45 | 59.82 |
| AMPV_B_400_14_LEFT | 2 | TCATGTTATGAAGGTGTGAGTTGC | 24 | 41.67 | 59.86 |
| AMPV_B_400_14_RIGHT | 2 | CATAGTAAGCTATGCCCACTACGA | 24 | 45.83 | 60.1 |
| AMPV_B_400_15_LEFT | 1 | GTCTAATGACTTACTGGACATAGAGGT | 27 | 40.74 | 60.21 |
| AMPV_B_400_15_RIGHT | 1 | TCTCTCCCTGCTCCTGATATGAG | 23 | 52.17 | 60.76 |
| AMPV_B_400_16_LEFT | 2 | AGTGCGGGCTAATTATATGCTGA | 23 | 43.48 | 60.25 |
| AMPV_B_400_16_RIGHT | 2 | TTCCCCTTCTGAACCAGCAGAA | 22 | 50 | 61.75 |
| AMPV_B_400_17_LEFT | 1 | ACAGTCTCAAGAGGCTACCTAAAGA | 25 | 44 | 61.14 |
| AMPV_B_400_17_RIGHT | 1 | GAGGATGTGCTTGATCCAAGGT | 22 | 50 | 60.54 |
| AMPV_B_400_18_LEFT | 2 | GCGGTTTACACATCTTACACTTAGT | 25 | 40 | 59.74 |
| AMPV_B_400_18_RIGHT | 2 | ACTGGTGGGTGAGATCGGTAAT | 22 | 50 | 61.08 |
| AMPV_B_400_19_LEFT | 1 | AAGGAATACTTGCCAGCTGAGG | 22 | 50 | 60.54 |
| AMPV_B_400_19_RIGHT | 1 | ACTTGTATGTATATATTCATATGCTGGTTGC | 31 | 32.26 | 61.08 |
| AMPV_B_400_20_LEFT | 2 | CTGGGCTCTGGTGTTGTTACAA | 22 | 50 | 60.93 |
| AMPV_B_400_20_RIGHT | 2 | TTGTTGTAGTCTTACCTCTTGTTGAAG | 27 | 37.04 | 59.99 |
| AMPV_B_400_21_LEFT | 1 | TGTGTGGACACTTATTGGGTGG | 22 | 50 | 60.67 |
| AMPV_B_400_21_RIGHT | 1 | GGAGGAGTTGATTTGCTGTTGG | 22 | 50 | 59.95 |
| AMPV_B_400_22_LEFT | 2 | TTTCTTCCCATGGGGTGAGTTG | 22 | 50 | 60.68 |
| AMPV_B_400_22_RIGHT | 2 | GAGTTGCTGTGCTGTGGGTTT | 21 | 52.38 | 61.73 |
| AMPV_B_400_23_LEFT | 1 | AACACCAGAGGCACCAGCAA | 20 | 55 | 62.3 |
| AMPV_B_400_23_RIGHT | 1 | CACAGATGCATCTCCTGGTCCT | 22 | 54.55 | 61.99 |
| AMPV_B_400_24_LEFT | 2 | GGTTCCAAAATGTTCCACTTATGGG | 25 | 44 | 60.95 |
| AMPV_B_400_24_RIGHT | 2 | GCTCAATGACAGGACTTGTGGT | 22 | 50 | 60.99 |
| AMPV_B_400_25_LEFT | 1 | AGCGAAACAAATGCAATTGGATCT | 24 | 37.5 | 60.64 |
| AMPV_B_400_25_RIGHT | 1 | TTCCAGGCACCTATAAGCTTGT | 22 | 45.45 | 59.61 |
| AMPV_B_400_26_LEFT | 2 | ACCTACCTGAATGGCTAGAGCA | 22 | 50 | 60.81 |
| AMPV_B_400_26_RIGHT | 2 | TCCAGAATCCTTCATTGCCAGT | 22 | 45.45 | 59.88 |
| AMPV_B_400_27_LEFT | 1 | GGAGCAAACTCGTTGGAGAACT | 22 | 50 | 60.99 |
| AMPV_B_400_27_RIGHT | 1 | ATGGCCTCCCTTTCTTCTACCA | 22 | 50 | 61.02 |
| AMPV_B_400_28_LEFT | 2 | AACGATATGTCCAATGCAGCAG | 22 | 45.45 | 59.56 |
| AMPV_B_400_28_RIGHT | 2 | AGGGGAGATTGCCTTGTCATTG | 22 | 50 | 60.81 |
| AMPV_B_400_29_LEFT | 1 | TGCTGAAGACTTCTTAGAGTTAGCA | 25 | 40 | 60.14 |
| AMPV_B_400_29_RIGHT | 1 | ACAGTTTTTCTGCAAGTATCTGCAC | 25 | 40 | 60.88 |
| AMPV_B_400_30_LEFT | 2 | TCTCTTTGACTGGAAAAGAAAGGGA | 25 | 40 | 60.49 |
| AMPV_B_400_30_RIGHT | 2 | TGGTGCATGCCTATAAGTGCAT | 22 | 45.45 | 60.61 |
| AMPV_B_400_31_LEFT | 1 | GCTCCATGGTACTCAGAGCCTA | 22 | 54.55 | 61.2 |
| AMPV_B_400_31_RIGHT | 1 | TGTGCCCTATGTCAGAATAAGCA | 23 | 43.48 | 60.06 |
| AMPV_B_400_32_LEFT | 2 | ACCTGTGAGGCTTATTGGTACTC | 23 | 47.83 | 60.06 |
| AMPV_B_400_32_RIGHT | 2 | CTGACTACCTGCTAAACTGTGTCT | 24 | 45.83 | 60.34 |
| AMPV_B_400_33_LEFT | 1 | GGGGGAATCATTTACTACAAGCTTG | 25 | 44 | 60.31 |
| AMPV_B_400_33_RIGHT | 1 | ACCCAATAGCCTGAGGATCTCT | 22 | 50 | 60.28 |
| AMPV_B_400_34_LEFT | 2 | AGGTTCTTCCTCACACTAATGAACA | 25 | 40 | 60.32 |
| AMPV_B_400_34_RIGHT | 2 | TGAGCAACATCATATGAACTGCTCT | 25 | 40 | 61.01 |
| AMPV_B_400_35_LEFT | 1 | AGTAGTTAACATGATAGCAGGTACCA | 26 | 38.46 | 59.83 |
| AMPV_B_400_35_RIGHT | 1 | TTTTCTCCTGCGTGCTAGATCC | 22 | 50 | 60.6 |
| AMPV_B_400_36_LEFT | 2 | AGAAGTTCTCGGCAGAAAAGACT | 23 | 43.48 | 60.18 |
| AMPV_B_400_36_RIGHT | 2 | CTCTCACTAAGTCTTTTGTTGATAGGG | 27 | 40.74 | 59.94 |
| AMPV_B_400_37_LEFT | 1 | GGCCGATGGAGTTTCCTTCATC | 22 | 54.55 | 61.5 |
| AMPV_B_400_37_RIGHT | 1 | TCTCAGTTGCAATTCTCTCTGCA | 23 | 43.48 | 60.5 |
| AMPV_B_400_38_LEFT | 2 | TGATCATATCAGCCTCACAATGCT | 24 | 41.67 | 60.53 |
| AMPV_B_400_38_RIGHT | 2 | TTGGTTCAAGGAAAACCTTGCTC | 23 | 43.48 | 60.12 |
| AMPV_B_400_39_LEFT | 1 | ACATTGTAGAAGACCCAATTGACAG | 25 | 40 | 59.67 |
| AMPV_B_400_39_RIGHT | 1 | GGTGTAACATCGGTTGGGGTAG | 22 | 54.55 | 60.86 |
| AMPV_B_400_40_LEFT | 2 | ATGCTATGACACGGCTGCTTAG | 22 | 50 | 60.98 |
| AMPV_B_400_40_RIGHT | 2 | TCAAGCCAGGATATTCACATGCT | 23 | 43.48 | 60.38 |
| AMPV_B_400_41_LEFT | 1 | GGTGGCAAAACTAGACCTCAGA | 22 | 50 | 60.14 |
| AMPV_B_400_41_RIGHT | 1 | CAGCATGTACACACTTGTCCCG | 22 | 54.55 | 61.93 |
| AMPV_B_400_42_LEFT | 2 | AGACCCTGATAACGTACTTTTATTAATCAT | 30 | 30 | 59.81 |
| AMPV_B_400_42_RIGHT | 2 | TGTCCAAGTGTGTTAGGGTTGA | 22 | 45.45 | 59.68 |
| AMPV_B_400_43_LEFT | 1 | AGTCTTTAGCCCCAGGGTTATCT | 23 | 47.83 | 60.83 |
| AMPV_B_400_43_RIGHT | 1 | ACCCCTTTTTCCTATTGGATTTTTAGT | 27 | 33.33 | 59.77 |
